# Genome-wide transcriptional analysis of super-embryogenic *Medicago truncatula *explant cultures

**DOI:** 10.1186/1471-2229-8-110

**Published:** 2008-10-27

**Authors:** Nijat Imin, Nicolas Goffard, Mahira Nizamidin, Barry G Rolfe

**Affiliations:** 1 Australian Research Council Centre of Excellence for Integrative Legume Research, Genomic Interactions Group, Research School of Biological Sciences, Australian National University, Canberra City, ACT 2601, Australia; 2 Institut Louis Malardé, GP Box 30, 98713 Papeete Tahiti, French Polynesia

## Abstract

**Background:**

The *Medicago truncatula *(*M. truncatula*) line 2HA has a 500-fold greater capacity to regenerate plants in culture by somatic embryogenesis than its wild type progenitor Jemalong. To understand the molecular basis for the regeneration capacity of this super-embryogenic line 2HA, using Affymetrix GeneChip^®^, we have compared transcriptomes of explant leaf cultures of these two lines that were grown on media containing the auxin NAA (1-naphthaleneacetic acid) and the cytokinin BAP (6-benzylaminopurine) for two weeks, an early time point for tissue culture proliferation.

**Results:**

Using Affymetrix GeneChip^®^, GCRMA normalisation and statistical analysis, we have shown that more than 196 and 49 probe sets were significantly (p < 0.05) up- or down-regulated respectively more than 2 fold in expression. We have utilised GeneBins, a database for classifying gene expression data to distinguish differentially displayed pathways among these two cultures which showed changes in number of biochemical pathways including carbon and flavonoid biosynthesis, phytohormone biosynthesis and signalling. The up-regulated genes in the embryogenic 2HA culture included nodulins, transporters, regulatory genes, embryogenesis related arabinogalactans and genes involved in redox homeostasis, the transition from vegetative growth to reproductive growth and cytokinin signalling. Down-regulated genes included protease inhibitors, wound-induced proteins, and genes involved in biosynthesis and signalling of phytohormones auxin, gibberellin and ethylene. These changes indicate essential differences between the super-embryogenic line 2HA and Jemalong not only in many aspects of biochemical pathways but also in their response to auxin and cytokinin. To validate the GeneChip results, we used quantitative real-time RT-PCR to examine the expression of the genes up-regulated in 2HA such as transposase, RNA-directed DNA polymerase, glycoside hydrolase, *RESPONSE REGULATOR 10*, *AGAMOUS-LIKE 20*, flower promoting factor 1, nodulin 3, fasciclin and lipoxygenase, and a down-regulated gene *ETHYLENE INSENSITIVE 3*, all of which positively correlated with the microarray data.

**Conclusion:**

We have described the differences in transcriptomes between the *M. truncatula *super-embryogenic line 2HA and its non-embryogenic progenitor Jemalong at an early time point. This data will facilitate the mapping of regulatory and metabolic networks involved in the gaining totipotency and regeneration capacity in *M. truncatula *and provides candidate genes for functional analysis.

## Background

Plants are well known for their extraordinary capacity to regenerate whole organisms from somatic cells. They often retain plasticity and have the capability to reverse the differentiation process and change their fate. The remarkable plasticity of plant cells is well exemplified by the capability of differentiated leaf cells to retain totipotency, the ability of a single cell to develop into a new organism [1]. This process is known as somatic or asexual embryogenesis (SE) whereby somatic cells differentiate into embryos and ultimately into plants via a series of characteristic morphological stages, particularly the later stages, which resemble the zygotic stages of development [2,3]. SE is the developmental restructuring of somatic cells towards the embryogenic pathway and forms the basis of cellular totipotency in higher plants [4,5]. Analyses of gene expression during somatic embryogenesis can provide information about the early stages of plant development [2]. Large-scale transcription analyses of embryogenesis have also been reported in several species [6-12]. Numerous genes have been identified as specifically expressed during somatic embryogenesis [13,14]. These genes include hormone responsive genes such as auxin inducible genes [15], late embryo abundant genes [16], calmodulin [17], calcium dependent/calmodulin-independent protein kinases [18], calmodulin-like protein kinases [19], somatic embryogenesis receptor-like kinase (*SERK*) genes [3,4,20], homeobox containing genes [21,22]; chitinases [23]; arabinogalactans [24], lipid transfer proteins [25], *WUSCHEL *[26] and *LEAFY COTYLEDON *genes [27,28], to name a few. As yet little is known about the induction and maintenance process of the genes involved in the SE processes, especially in the acquisition of totipotency of somatic cells. Avivi *et al*. has shown that that acquisition of pluripotentiality involves changes in DNA methylation pattern and reorganisation of specific chromosomal subdomains. These changes lead to activation of silent genes such as plant specific *NAC *(no apical meristem-like) genes and *VIP1*, a gene encoding b-Zip nuclear protein that involved in acquisition or maintenance of pluripotentiality [29]. Several researchers have sought to identify the very early plant cells in the explant cell population that are competent to be committed to differentiation pathways. Using the *SERK *gene as a marker during the examination of either carrot hypocotyls explants [3], immature zygotic embryos of sunflower [20], leaf explants of *Dactylis glomerata *L. (Poaceae) [30], or developing ovules and embryos of *Arabidopsis *[31]. *SERK *gene is expressed early in a small sub-population of cells which are competent to form embryogenic cells [3]. Over-expression of the *AtSERK1 *gene in *Arabidopsis *cultures was shown to induce somatic embryo formation [31]. Similarly, the over-expression of a transcription factor called *BABY BOOM *(*BBM*) that shows similarity to the AP2/EREPB multigene family of transcription factors [32] under the control of the 35S promoter in transgenic plants induced ectopic spontaneous somatic embryos and cotyledon-like structures on *Arabidopsis *and Brassica seedlings. The *BBM *gene was originally isolated because it represented a gene that was expressed early in the initiation of the differentiation of embryo development from immature pollen grains of *Brassica napus *(microspore embryogenesis) and appeared to be involved in the conversion from vegetative to embryonic growth [32].

Legumes in general have proven recalcitrant at *de novo *regeneration in vitro [33]. In *Medicago truncatula*, leaf explants as well as protoplasts can form calli and subsequently the generation of embryos and then the development of plants [34]. Depending on the plant system, auxin and/or cytokinin are required to enable embryogenesis to occur in culture [3,30,31,34]. In *Medicago truncatula*, Nolan *et al*. found that embryogenesis required both auxin and cytokinin addition, although some embryos could form on cytokinin alone [4]. In the leaf explant tissue culture system, there is an advantage of being able to manipulate the type of differentiating cells observed by changing the phytohormones added to the culturing media [3,4,20], and embryos are initiated more rapidly in 4–6 weeks. This meristematic system has ideal attributes: the regenerative capacity of the mutant line 2HA, which is 500 fold more embryogenic than its isogenic line Jemalong [34,35]. When both *M. truncatula *cultivar (cv) Jemalong and 2HA explant tissues are cultured in medium with addition of auxin and cytokinin, the 2HA explants form embryos. Generally cv Jemalong does not form embryos but does produce early vascularisation in the calli. The pasture legume *M. truncatula *(Australian barrel medic) is one of the model systems for the analysis of the unique biological and fundamental processes governing legume biology. Recent genomic tools, advanced DNA sequencing programs, EST libraries and Medicago GeneChip^® ^have been developed for this legume and we previously have established proteome reference maps for *M. truncatula *somatic embryogenesis cultures and compared the proteome of the super-embryogenic line 2HA with that of non-embryogenic progenitor Jemalong [5,36]. In this study, we have used leaf explant tissue cultures of 2HA and Jemalong to investigate gene expression profiles and their changes during the early stage of regeneration and to identify key regulatory factors and the early markers of cell competency for regeneration.

## Results

### Transcriptomic analysis of the super-embryogenic line 2HA and its progenitor Jemalong

**Figure 1:**
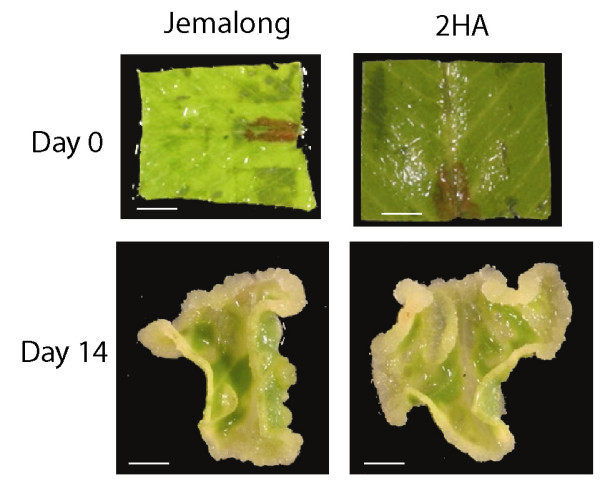
**Explant leaf in-vitro tissue culture of *M. truncatula *2HA and its progenitor Jemalong**. They were grown on media that contained 10 μM NAA (1-naphthaleneacetic acid) and 4 μM BAP (6-benzylaminopurine). Bar = 2.5 mm.

The *M. truncatula *line 2HA has a 500 fold greater capacity to regenerate plants in culture by somatic embryogenesis than its progenitor Jemalong. Figure 1 shows explant leaf tissue cultures of *M. truncatula *super-embryogenic seed line 2HA and its progenitor Jemalong at day 0 and day 14. They were grown on medium containing 10 μM NAA and 4 μM BAP. At two weeks, callus cells start to proliferate and there are no morphological differences can be seen between the 2HA and Jemalong. Thus, two weeks provides an early time point to compare proliferating cultures of these two lines. The first appearance of embryos in 2HA occurs after five weeks of culture, but not in Jemalong [34,36]. To investigate gene expression profiles and their changes during the early stage of regeneration, we profiled and compared the transcriptomes of 2HA and Jemalong, by extracting total RNA from three independently grown two-week old cultures and analysing them on Affymetrix Medicago genome arrays. An average of 46% (24,080 ± 572 probe sets) of the over 52,000 plant gene probe sets of the Medicago Genome Array GeneChip produced 'present' calls when hybridised with biotin-labelled cRNA from *M. truncatula *tissue culture similar with early reports in root and leaf samples [37,38]. Following normalisation with GCRMA, we identified only 196 probe sets (0.38% of the total probe sets) that are at least 2.0 fold over-expressed (p < 0.05) in the super-embryogenic line 2HA and only 49 probe sets (0.09% of the total probe sets) that are over-expressed (p < 0.05) in the non-embryogenic Jemalong. The vast majority of probe sets (over 99.5%) did not show any significant change between the cultures. The choice of 2-fold threshold is somewhat arbitrary but in combination with student t test and its associated p values, it is intended to emphasize on major changes. We also performed the Significance Analysis of Microarrays (SAM) two-class unpaired analysis in order to identify a more extensive list of differentially expressed genes [43]. We found that 560 and 107 probes were up- or down-regulated respectively (additional file 1). There were significant similarities between 2-fold cut-off method and SAM two-class unpaired analysis. One hundred ninety five probe sets were up-regulated in the embryogenic culture by both analyses while 38 probe sets were down-regulated (additional file 2). The full data set has been deposited in the Gene Expression Omnibus database as accession GSE8131 and the normalised data set is available in the additional file 3.

### Array verification

Quantitative real-time RT-PCR was used to confirm the level of expression of 10 transcripts from the array (Table 1). For all probe sets tested, the expression ratios displayed the same pattern of expression as the array normalised data but with amplified fold-changes. For instance, probe set Mtr.10439.1.S1_at (*MtEIN3*) showed down-regulation by real-time RT-PCR in the embryogenic culture, consistent with the array data. In average, the fold changes in RT-PCR data were approximately three times higher than that of array data indicating amplification of fold changes by sensitive real-time RT-PCR analysis. However, the fold changes were much closer to array un-normalised data (3.4 times higher than the normalised array data; data not shown) indicating normalisation may considerably reduce the signal differences. The functional significance of the transcripts validated by qRT-PCR is discussed in more details below.

**Table 1:** Comparison of real-time RT-PCR and microarray results for selected genes

**Probe ID**	**Annotation**	**Microarray (log2)**	**RT-PCR (log2)**
Mtr.47631.1.S1_s_at	Transposase	1.43 ± 0.28	4.22 ± 0.53
Mtr.15107.1.S1_at	RNA-directed DNA polymerase	1.37 ± 0.14	4.39 ± 0.89
Mtr.45925.1.S1_s_at	GH	1.32 ± 0.43	6.92 ± 0.58
Mtr.43735.1.S1_at	MtRR1	1.10 ± 0.30	2.77 ± 0.17
Mtr.47174.1.S1_at	AGL20	1.06 ± 0.10	4.84 ± 0.16
Mtr.41073.1.S1_at	FPF1	1.32 ± 0.14	3.33 ± 0.37
Mtr.8427.1.S1_at	LipOx	1.19 ± 0.34	3.10 ± 0.53
Mtr.10439.1.S1_at	EIN3	-1.41 ± 0.11	-2.76 ± 0.09
Mtr.8585.1.S1_at	MtN3	1.71 ± 0.08	6.24 ± 0.28
Mtr.18380.1.S1_at	Fasciclin	1.52 ± 0.02	3.9 ± 0.03

These *M. truncatula *probe set IDs are from the Medicago GeneChip. *GH, Glycoside hydrolase (note this gene also incorrectly annotated as Regulator of chromosome condensation on the array*); *RR*, response regulator; *AGL20*, agamous-like 20; *FPF*, flower promoting factor; *LipOx*, lipoxygenase; *EIN*, ethylene-insensitive; *MtN3*, *M. truncatula *nodulin3. Values shown are ratios of the means of three independent measurements from microarray data or real-time RT-PCR data. Note the Log_2 _changes are given rather than fold changes. Standard deviations are given as ± values.

### Functional classification of differentially expressed probe sets

**Figure 2:**
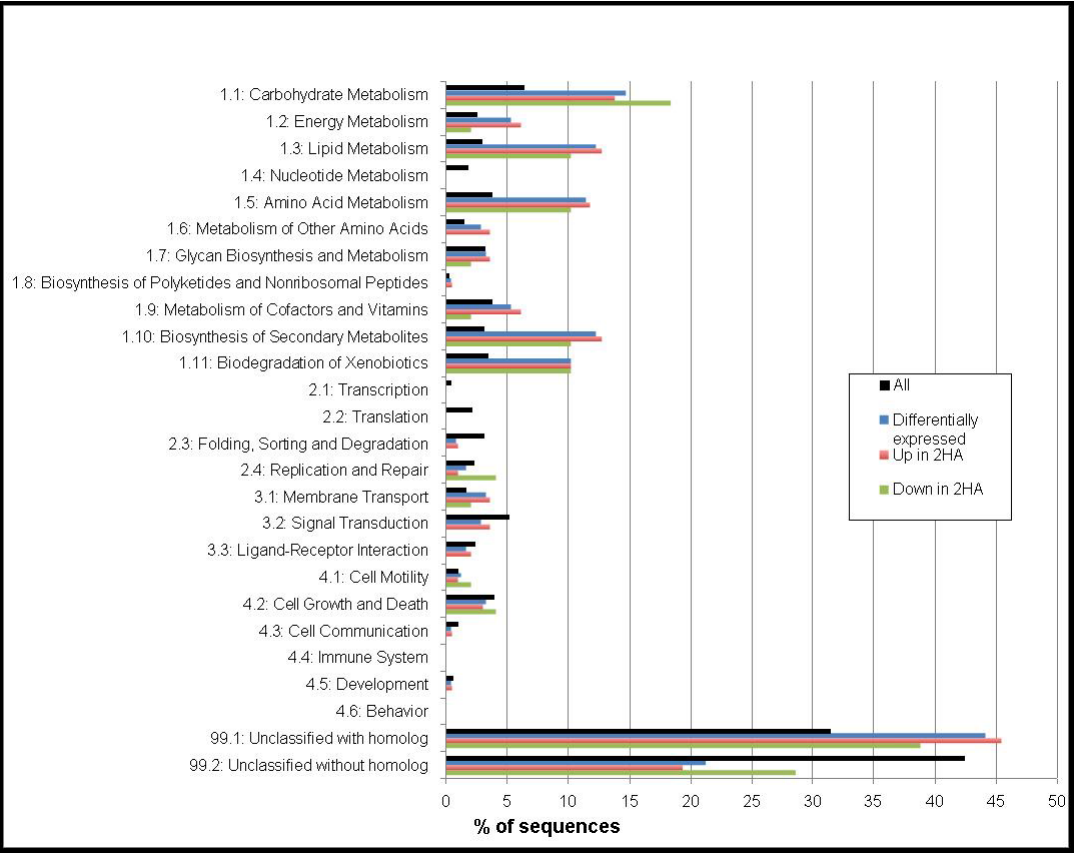
**Classification of expression changes with GeneBins**. Differentially, up- and down-regulated probe sets in the embryogenic culture when compared to that of Jemalong are represented by blue, red and green columns respectively. Classification of all of the *M. truncatula *probe sets are represented by black columns. GeneBins classification of probe sets with changes in expression that are significant (P ≤ 0.05) at 2.0 fold. See Methods for the details of classification.

The Medicago genome array does not incorporate the entire *M. truncatula *genome, it was created based on an incomplete genome sequence and ESTs from the *Medicago truncatula *Gene Index (MtGI). We have noted the inclusion of probe sets for IMGAG gene predictions and the corresponding EST leading to a duplication of data, and the absence of some consensus ESTs from MtGI available at the time the chip was made and also incorrect annotation of some genes in both IMGAG and MtGI (data not shown). Annotation of the probe sets on the Genome array also varies widely in quality. To interpret the gene expression data better, we have used GeneBins to provide hierarchical functional classification modelled on KEGG ontology [39,40]. This analysis made it apparent that the metabolism seems to be different between the embryogenic and the non-embryogenic *M. truncatula *cultures (Figure 2). About 35% percent of differentially expressed probe sets could be assigned a functional classification with GeneBins; of note 14.7% (p = 1.6E-4), 12.2% (p = 2.5E-8) and 12.2% (p = 3.7E-8) of transcripts differentially expressed are involved in carbohydrate metabolism, lipid metabolism and the biosynthesis of secondary metabolites respectively. Around 21% of differentially expressed transcripts have no homolog, however by far the largest class of probe sets that had significantly altered expression in our analysis were unclassified with a homolog (44%). This result led us to use other bioinformatics strategies to annotate the probe sets on the genome array.

To further refine the functional classification and annotation of metabolic probe sets on the Medicago genome array we used PathExpress [41]. Using this database we were able to identify statistically significant over-representation of metabolic pathways in the embryogenic and non-embryogenic cultures as shown in Table 2. Five metabolic pathways are significantly over-represented in the embryogenic cultures. They are: (1) Sphingolipid (major component of the plasma membrane, tonoplast, and other endo-membranes of plant cells) metabolism [represented by over-expression of cytochrome P450s 86A1 (Mtr.39593.1.S1_at), 94A1 (Mtr.51652.1.S1_at), 71A1 (Mtr.33655.1.S1_at), 90D2 (Mtr.27152.1.S1_at), phosphatase phospho1 (Mtr.10566.1.S1_at) and beta-galactosidase (Mtr.43150.1.S1_at)]; (2) Stilbene, coumarine and lignin biosynthesis [represented by over-expression of peroxidases (Mtr.7245.1.S1_at, Mtr.37599.1.S1_at, Mtr.38167.1.S1_at, Mtr.51089.1.S1_at, Mtr.9899.1.S1_at, Mtr.10375.1.S1_at, Mtr.38635.1.S1_at), caffeic acid 3-O-methyltransferase (Mtr.43098.1.S1_at) and cytochrome P450s 86A1 (Mtr.39593.1.S1_at), 94A1 (Mtr.51652.1.S1_at), 90D2 (Mtr.27152.1.S1_at) and 71A1 (Mtr.33655.1.S1_at)]; (3) Flavonoid biosynthesis (represented by over-expression of leucoanthocyanidin dioxygenase (Mtr.40209.1.S1_at), flavonol 3-O-glucosyltransferase (Mtr.9255.1.S1_at) and 3-ketoacyl-CoA synthase 12 (Mtr.49305.1.S1_at); (4) Riboflavin metabolism [tartrate-resistant acid phosphatase type 5 (Mtr.44281.1.S1_at) and phosphatase phospho1 (Mtr.10566.1.S1_at)] and (5) biosynthesis of 12-, 14- and 16-membered macrolides [quinone oxidoreductase (Mtr.44591.1.S1_at)]. Two pathways are over-represented in the non-embryogenic cultures. They are: (1) Ascorbate and aldarate metabolism [represented by over-expression of ascorbate oxidase (Mtr.9478.1.S1_at), cytochrome P450s 90B1 (Mtr.19623.1.S1_at), 71D9 (Mtr.23217.1.S1_at and Mtr.47492.1.S1_at) and 94A1 (Mtr.45741.1.S1_at)]. (2) Biosynthesis of 12-, 14- and 16-membered macrolides [represented by auxin inducible quinone oxidoreductase (Mtr.18492.1.S1_at)].

**Table 2:** Potential metabolic differences in embryogenic and non-embryogenic cultures.

**Embryogenic 2HA culture**			
***Pathways***	***No. E.C. numbers in genome array pathway***	***No. E.C. numbers expressed ≥ 2.0 fold***	***P value***
Sphingolipid metabolism	11	3	9.60E-03
Stilbene, coumarine and lignin biosynthesis	12	3	1.24E-02
Flavonoid biosynthesis	14	3	1.94E-02
Riboflavin metabolism	7	2	3.33E-02
Biosynthesis of 12-, 14- and 16-membered macrolides	1	1	4.34E-02
**Non-embryogenic Jemalong culture**			
Ascorbate and aldarate metabolism	10	2	2.73E-03
Biosynthesis of 12-, 14- and 16-membered macrolides	1	1	8.6E-02

Potential metabolic pathways significantly over-represented (p ≤ 0.05) amongst differentially expressed probe sets at 2.0 fold as determined by PathExpress.

We also annotated the array by comparing the data set with the Arabidopsis Gene Family Information database maintained by the Arabidopsis Information Resource [42]. As of April 2007 the database contained 996 gene families and 8,331 genes. Using Blast, we were able to classify 3,159 Medicago probe sets into these families. Forty one and ten of the over expressed probe sets from the embryogenic and non-embryogenic cultures respectively were classified in the gene families. Two cytochrome P450 families (CYP94C, p = 0.016 and CYP90B, p = 0.047) were significantly over-represented in the non-embryogenic line Jemalong (Additional file 4). Finally, transcription factors (TFs) on the Genome array were predicted by homology relationship based on the Database of Arabidopsis Transcription Factors [43]. This analysis showed that 2,323 probe sets on the Genome array have sequence homology to described plant TFs. Twenty one predicted TFs were up-regulated in the embryogenic line 2HA cultures and six TFs were up-regulated in the non-embryogenic Jemalong cultures (Table 4 and Additional file 5). The families represented in the embryogenic cultures are the basic/helix-loop-helix (bHLH), zinc finger domain TFs C2C2-co-like and C2C2-DOF, response regulators (GARP-ARR-B), GRAS domain containing TFs (GRAS), MADS-box TFs (MADS) and MYB DNA-binding domain TFs (MYB). The TF families represented in the non-embryogenic cultures are APETALA 2 and ethylene-responsive element binding proteins (AP2/EREBP), auxin-responsive protein/indoleacetic acid-induced protein (AUX/IAA) and ETHYLENE INSENSITIVE 3 (EIN3). With the exception of bHLH and zinc finger containing TFs, the TF gene families are plant specific. We confirmed the expression of several TFs betweens the cultures of two lines using qRT-PCR (Table 1).

### Phytohormone biosynthesis and signalling

Although GeneBins and PathExpress are valuable tools to identify gene classes and molecular pathways in general, they are not designed to identify plant specific pathways such as phytohormone biosynthesis and signalling. Thus, we manually analysed the differentially displayed genes involved in these processes. We have identified two probe sets Mtr.30770.1.S1_at & Mtr.10439.1.S1_at that are homologues to Arabidopsis *ETHYLENE INSENSITIVE3 *(*EIN3*). These two probe sets were down-regulated 2.6 fold and 1.8 fold respectively, in the embryogenic 2HA cultures. Similarly, a probe set for GA2-oxidase (GA2ox) (Mtr.33914.1.S1_at) and a probe set (Mtr.22904.1.S1_s_at) for an *IAA*/*AUX *gene was down-regulated in the embryogenic 2HA cultures. In contrast, a response regulator (MtRR1, Mtr.43735.1.S1_at) was up-regulated the embryogenic 2HA cultures. This was confirmed by real-time RT-PCR (Table 1).

### Comparison of gene expression between the embryogenic cultures and seed development

To identify common genes expressed between embryogenic cultures (somatic embryogenesis) and developing seeds (zygotic embryogenesis), we have compared our data to that from the Medicago Expression Atlas [44]. We have chosen developing seeds at 10 days after pollination since this is the earliest time point available for seed development in the Atlas and contrasted it to leaf. A total of 12,954 probe sets showed differentially display between the developing seed at ten days after pollination and leaf samples. Over 6,800 probe sets were up-regulated in the developing seeds at least two fold (P < 0.05), of which 14 were also up-regulated in the embryogenic cultures when compared to non-embryogenic cultures (additional files 6 and 7). These include a basic helix-loop-helix (bHLH) transcription factor (Mtr.51379.1.S1_at), MtRR1 (response regulator, Mtr.43735.1.S1_at), a putative phosphatase (Mtr.10566.1.S1_at), an E1-E2 type ATPase (Mtr.26397.1.S1_s_at), a serine carboxypeptidase (Mtr.10023.1.S1_at), a GDSL-motif lipase (Mtr.13241.1.S1_at), a peroxidase (Mtr.10375.1.S1_at), two nodulins (Mtr.11717.1.S1_at and Mtr.41025.1.S1_at), a fatty acid elongase (Mtr.49305.1.S1_at) and four unknown proteins (Mtr.35655.1.S1_at, Mtr.18491.1.S1_at, Mtr.38330.1.S1_at and Mtr.14656.1.S1_at). Over 6,000 probe sets were down-regulated in the developing seeds at least two fold (P < 0.05), of which 6 were also down-regulated in the embryogenic cultures when compared to non-embryogenic cultures (additional files 6 and 7). these include transcription factor EIL1 (Mtr.10439.1.S1_at), a H^+^-transporting ATPase Mtr.5635.1.S1_at), Snakin-like cysteine rich protein (Mtr.12742.1.S1_at), a patatin-like phospholipase (Mtr.37859.1.S1_at), a thaumatin-like protein (Mtr.33691.1.S1_at) and a hypothetical protein (Mtr.43627.1.S1_at). In brief, we have identified a small number probe sets that were either up- or down-regulated in both the embryogenic cultures and the developing seeds. These include transcription factors such as response regulator MtRR1 and EIN3, nodulins and unknown proteins (additional file 6). Further investigation of these proteins will shed light on the similarities between somatic and zygotic embryogenesis.

### Comparison between the array and proteomics

We also compared our array data with the proteome data obtained for the explant leaf cultures of 2HA and Jemalong [36]. 16 protein spots were reportedly identified as differentially displayed proteins between the explant leaf cultures of 2HA and Jemalong after 2, 5 and 8 weeks of culture. Although all of the corresponding genes were present on the array, none of them showed differential display when used 2 fold cut-off and student t test (data not shown). Thus, we were not able to find any correlation between transcriptomics and proteomics of the explant leaf cultures of 2HA and Jemalong. This probably due to the fact that only a very limited number of differentially displayed proteins were identified by proteomics, most of which showed differential display only at the later stages of culture (5 and 8 weeks of culture) but not at the early stage (at two weeks) at which this microarray analysis was focused on.

## Discussion

During the initial phases of organogenesis somatic cells progress through a series of events referred to as differentiation, competence acquisition, induction and determination [20]. Most in vitro cultures require auxin in the medium to initiate these steps while sunflower immature zygotic embryos do not. They do, however require cytokinin to induce somatic embryogenesis [20,45]. Working with immature zygotic embryos of sunflowers, Thomas *et al*. showed that the time of exposure to a specific medium was fundamental to the commitment to a particular morphogenic pathway [20]. This period was described as embryogenic competence during the morphogenic induction. The period lasted for three days when the commitment could be reversed by changing the medium. However, after four days it could not be altered and thus an irreversible step was taken within the competent cells toward a particular organogenesis pathway. Seven days of pre-treatment with auxin can interrupt somatic embryo formation in *M. truncatula *[46]. And at two weeks, the explant leaves start to proliferate. Thus, we reasoned that comparing transcriptomes of two-week old tissue cultures of super-embryogenic 2HA and its non-embryogenic progenitor Jemalong would reveal important genes involved in early steps of regeneration and acquiring totipotency. The transcriptomic analysis has revealed changes in gene expression between the super-embryogenic line and the non-embryogenic line of *M. truncatula*, although the vast majority of probe sets (over 99.5%) did not show any significant change between the cultures. The differentially expressed genes include genes involved in various metabolic pathways, flavonoid biosynthesis, hormone biosynthesis and signalling and genes involved in gene regulation.

### Arabinogalactan proteins

We have identified five probe sets (Mtr.18380.1.S1_at, Mtr.10992.1.S1_at, Mtr.17361.1.S1_at, Mtr.51607.1.S1_at and Mtr.50900.1.S1_at) belonging to Beta-Ig-H3 fasciclin-like arabinogalactan proteins (AGPs) that are up-regulated in the embryogenic cultures of 2HA at least two fold. AGPs are implicated in diverse developmental roles including somatic embryogenesis [47] although their exact functions remain unclear. AGPs containing N-acetylglucosamine can be a substrate for chitinase [48] leading to the release of oligosaccharide signal molecules that are necessary to induce somatic embryo formation [49]. The involvement of extracellular signal molecules in somatic embryogenesis has been reported in several plant species. It was shown that when non-embryogenic cultures were treated with growth medium conditioned by super-embryogenic cultures, the cultures became embryogenic [50]. Several components in the conditioned growth medium have been found to promote somatic embryogenesis. These components include chitinases [23] and AGPs [51-54]. It has been suggested that oligosaccharides released from AGPs by a chitinase act as signal molecules stimulating somatic embryogenesis [55]. However, the role of AGPs in the induction of somatic embryogenesis in *M. truncatula *is not understood yet.

### Genes involved in transition from vegetative growth to reproductive growth

We have identified an Arabidopsis ortholog of *FLOWERING PROMOTING FACTOR1 *(*AtFPF1*) that was 2.3 fold up-regulated in 2HA (Mtr.41073.1.S1_at). *AtFPF1 *is one of the important genes involved in the genetic control of flowering time in Arabidopsis. It is expressed in apical meristems immediately after photoperiodic induction of flowering in long-day plants, which flower only when exposed to long days [56]. During the transition to flowering, the FPF1 gene is expressed at the same time as *LEAFY *and earlier than *APETALA1*, two key unrelated TFs in flower initiation. FPF1 modulates the acquisition of competence to flower in the apical meristem. Over-expression of FPF1 leads to early flowering in Arabidopsis [57]. Similar results were also reported in tobacco [58]. However in rice, it has been shown that it also plays a role in the initiation of adventitious roots [59,60] and it has been reported that the same gene was induced by salt treatments in *M. truncatula *roots and may contribute to the reacquisition of root growth, notably through the emergence of lateral roots [61]. Another flowering promoting gene that was up-regulated (2.3 fold) in 2HA is *AGAMOUS-LIKE 20 *(*AGL20*, also known as *SUPPRESSOR OF OVEREXPRESSION OF CO 1 *or *SOC1*, Mtr.47174.1.S1_at) encodes a MADS box TF. In Arabidopsis, its ortholog was identified as a gene downstream of another MADS box TF FLC [62]. Activation of *AGL20 *causes early flowering despite strong expression of FLC, and knock out of *AGL20 *causes late flowering, suggesting that it is a flowering activator [62]. AGL20 is positively regulated by the long day pathway through CO, and negatively regulated by the autonomous/vernalisation pathway through FLC [62,63]. Since expression of *AGL20 *is regulated by signals from more than one flowering pathway it is referred to as a floral pathway integrator [64,65]. These genes function in 'cascades' within four promotive pathways, the 'photoperiodic', 'autonomous', 'vernalisation', and 'gibberellin' pathways, which all converge on the 'integrator' genes *AGL20 *(*SOC1*) and *FLOWERING LOCUS T *(*FT*) [66]. It has been shown that FLC directly interacts with the *AGL20 *and *FT *genes in vivo [67]. Probe set Mtr.7513.1.S1_at was up-regulated in 2HA and encodes a CONSTANS-like TF that are ortholog of At1g25440, which displayed root-specific expression [68] and are strongly repressed in N starvation [69] suggesting biological functions beyond promoting flowering.

Thus, we have identified three genes that were up-regulated in 2HA have similarities to the genes involved in transition from vegetative growth to reproductive growth, suggesting that initiation of both reproductive growth and regeneration share similar molecular processes.

### Nodulins

We identified eight genes classified as nodulins including early nodulin 75 (Mtr.38422.1.S1_at), MtN3s (Mtr.8585.1.S1_at & Mtr.11146.1.S1_at), MtN13 (Mtr.33137.1.S1_s_at & Mtr.37852.1.S1_at), nodulin 26 (Mtr.36842.1.S1_s_at) and other nodulins Mtr.43745.1.S1_at & Mtr.43508.1.S1_at). MtN3 protein contains MtN3 and saliva related transmembrane protein domain (Mtr.8585.1.S1_at & Mtr.11146.1.S1_at) and reported to be induced during nodulation in *M. truncatula *[70]. It has been shown in *ascidian Ciona intestinalis *that a gene encoding an MtN3/saliva family transmembrane protein is essential for tissue differentiation during embryogenesis [71]. MtN13, a homologue of plant defence proteins (Pathogenesis-related protein Bet v I family) has been reported to be nodulation/symbiosis-specific in *M. truncatula *[70]. Nod26, a member of plant aquaporins, also has been shown to be involved in nodulation [72,73]. Another non-nodulin proteins that has shown to be involved in nodule development is cycloartenol synthase [70]. We have detected the same gene (Mtr.4710.1.S1_s_at) highly up-regulated in the embryogenic line 2HA. These indicate that several genes expressed during nodule formation also expressed during regeneration in *M. truncatula*.

### Phytohormone biosynthesis and signalling

Two probe sets Mtr.10439.1.S1_at & Mtr.30770.1.S1_at that are homologues to Arabidopsis *ETHYLENE INSENSITIVE3 *(*EIN3*) were down-regulated 2.6 fold and 1.8 fold respectively, in the embryogenic line 2HA. The probe set Mtr.10439.1.S1_at was also down-regulated in the developing seeds at 10 days after pollination when compared to leaf samples, indicating some similarities between somatic and zygotic embryogenesis. EIN3 acts as a positive regulator at the most downstream position of the ethylene signal transduction pathway [74]. *EIN3 *encodes a transcription factor that belongs to a small family that includes EIN3 and various EIN3-like (EIL) proteins in Arabidopsis and it works downstream of EIN2 [74] and upstream of AtERF1, an early ethylene responsive gene [75]. Recently, Achard *et al*. has shown that activated ethylene signalling reduces bioactive Gibberellin (GA) levels and enhances the accumulation of DELLAs, and ethylene acts on DELLAs via the CTR1-dependent ethylene response pathway, most likely downstream of the transcriptional regulator EIN3. Ethylene-enhanced DELLA accumulation in turn delays flowering via repression of the floral meristem-identity genes *LEAFY *and *AGL20 *(*SOC1*), establishing a link between the CTR1/EIN3-dependent ethylene and GA-DELLA signalling pathways [76].

We have observed that a probe set for GA2-oxidase (GA2ox) (Mtr.33914.1.S1_at) was up-regulated in the non-embryogenic Jemalong cultures. GA has been implied to have an role in somatic embryogenesis in carrots [77], in Arabidopsis [78] and in Japanese cedar [79]. GA2ox, introduces a hydroxyl group at the 2β position, inactivating the GA molecule so that it cannot be converted into active forms [80,81]. These indicate that there is a reduction in active GA in this the non-embryogenic line Jemalong. However, the measuring of active GA contents in these lines is required to confirm such indication. It has been shown in Arabidopsis that *AGL20 *(or *SOC1*) is induced by GA [82] and we found *AGL20 *(*SOC1*, Mtr.47174.1.S1_at) to be up-regulated in the embryogenic line 2HA. The up-regulation of *AGL20 *correlates well with the up-regulation of GA2ox and down-regulation of *EIN3 *in the embryogenic line. Thus, our findings suggest that GA and ethylene may be involved in the acquisition of regeneration capacity in *M. truncatula *and indicate that AGL20 may be a key regulator that links GA and ethylene signalling.

We have identified a probe set (Mtr.22904.1.S1_s_at) for an *IAA*/*AUX *gene that was down-regulated in the embryogenic cultures. The corresponding gene is an ortholog of Arabidopsis *IAA20 *(AT2G46990). In Arabidopsis, IAA20 protein is long-lived and its longevity was not influenced by auxin suggesting they may play a novel role in auxin signalling [83]. We previously have shown that auxin (1-naphthaleneacetic acid) pre-incubation explant leaf tissues can irreversibly interrupt somatic embryo formation in the *M. truncatula *embryogenic line 2HA [46]. Thus, up-regulation of *IAA20 *ortholog in *M. truncatula *supports motion that the prolonged auxin signalling may have adverse effect on embryo formation.

Proliferation of undifferentiated callus tissue, greening, and the formation of shoot structures are all cytokinin-dependent processes. We have identified a response regulator (MtRR1, Mtr.43735.1.S1_at) that is up-regulated in the embryogenic cultures. This probe set was also up-regulated in the developing seeds at 10 days after pollination when compared to leaf samples, indicating some similarities between somatic and zygotic embryogenesis. MtRR1 is an ortholog of Arabidopsis ARR10 (RESPONSE REGULATOR 10; At4g31920) that belongs to B-type response regulators. It was reported that this gene is induced early in *M. truncatula *roots during the symbiotic interaction with *Sinorhizobium meliloti *[84]. There are other probe sets for the genes involved in cytokinin biosynthesis and signalling. However, these were not changed between the two cultures. For instance, there are two probe sets for adenylate isopentenyltransferases (cytokinin synthases, Mtr.31420.1.S1_at & Mtr.12113.1.S1_at) in the array and both probe sets did not expressed in both cultures. In contrast, *Cytokinin Response 1*, (*CRE1*, Mtr.12088.1.S1_at) [84] and other cytokinin inducible genes cyclin D3 (Mtr.35281.1.S1_at and Mtr.41123.1.S1_at), *KNAT *(Mtr.8842.1.S1_at), *SHOOT MERISTEMLESS *(Mtr.13772.1.S1_at) and type A response regulators (cytokinin-inducible) (Mtr.5343.1.S1_s_at, Mtr.32159.1.S1_at, Mtr.5335.1.S1_at, Mtr.43919.1.S1_at, Mtr.31738.1.S1_at and Mtr.174.1.S1_at) were also highly expressed in both cultures. These indicate that there are some differences between the embryogenic line 2HA and the non-embryogenic line Jemalong in respond to cytokinin and MtRR1 may be an important regulator in the acquisition of regeneration capacity in *M. truncatula*.

## Conclusion

We have described differences in transcriptomes between the *M. truncatula *super-embryogenic line 2HA and its non-embryogenic progenitor Jemalong. Notably they include significant variations in carbon and flavonoid metabolism, phytohormone biosynthesis and signalling, cell to cell communication and gene regulation. This data will facilitate the mapping of regulatory and metabolic networks involved in the acquisition of regeneration capacity of the embryogenic lines such as 2HA, and may lead to a better understanding of totipotency in *M. truncatula *and other legume species.

## Methods

### Plant materials, growth and tissue culture

*M. truncatula *cv Jemalong seed line 2HA and its progenitor Jemalong was used for the plant growth explant tissue culture as described [36,85]. Seeds of *M. truncatula *cv Jemalong were obtained from Professor Ray Rose (University of Newcastle, NSW, Australia). Plants were grown under controlled growth cabinet conditions with 12 hr photoperiod at 150 μmol m^-2 ^s^-1 ^with a day temperature of 23°C and a night temperature of 19°C and a relative humidity of 80%. The basal medium used for the explant leaf culture was P4, which is based on Gamborg's B5 medium as described [86]. In the usual culture procedure, leaf explants were plated onto P4 medium containing 10 μM NAA (1-naphthaleneacetic acid, Sigma-Aldrich, St. Louis, MO, USA) and 4 ╀M BAP (6-benzylaminopurine, Sigma-Aldrich). Cultures were incubated in the dark at 28°C.

### DNA microarray analysis

The Affymetrix Medicago GeneChip (Affymetrix, Santa Clara, CA, USA) contained 61,200 probe sets: 32,167 *M. truncatula *EST-based and chloroplast gene-based probe sets (TIGR Gene Index version 8, Jan., 2005, 36,878 unique sequences); 18,733 *M. truncatula *IMGAG (International Medicago Genome Annotation Group) and phase 2/3 BAC prediction-based probe sets; 1,896 *M. sativa *EST/mRNA based probe sets; 8,305 *Sinorhizobium meliloti *gene prediction-based probe sets.

### RNA isolation, hybridisation and data pre-processing

Total RNA was extracted and purified from the proliferating leaf explant cultures of *M. truncatula *line 2HA and Jemalong using the Qiagen RNeasy plant mini kit (Qiagen, Valencia, CA, USA). Total RNA was quantified using a NanoDrop ND-1000 Spectrophotometer; RNA with an absorbance A260/A280ratio >2.0 was quality tested using the Agilent 2100 Bioanalyzer. Preparation of cRNA, hybridisation, and scanning of the Test3 arrays and Medicago GeneChip^® ^were performed according to the manufacturer's protocol (Affymetrix, Santa Clara, CA, USA) (at the Biomolecular Resource Facility, JCSMR, ANU). Briefly, double-stranded cDNA was synthesised from 5 to 8 μg of each RNA sample via oligo T7-(dT)24 primer-mediated reverse transcription. Biotin-labelled cRNA was generated using the Enzo BioArray kit (Affymetrix), purified using RNeasy spin columns (Qiagen), and then quantified by spectrophotometer. Fifteen to 20 μg of each biotin-labelled fragmented cRNA sample was used to prepare 300 μL of hybridisation mixture. Aliquots of each sample (100 μL) were hybridised onto Test3 arrays to check the quality of the samples prior to hybridisation (200 μL) onto the Medicago genome arrays. The arrays were washed with optimised wash protocols, stained with strepdavidin/phycoerythrin followed by antibody amplification, and scanned with the Agilent GeneArray Scanner (Affymetrix).

### Data pre-processing

Raw Affymetrix data (cel files) were normalised with the GCRMA (GC content – Robust Multi-Array Average) algorithm (ver. 2.2.0) including quantile normalisation and variance stabilisation [87], using the Affymetrix package of the bioconductor software [88]. The normalised average of the replicates was then log transformed in base 2 to reduce the proportional relationship between random error and signal intensity. Differentially expressed probe sets were identified by evaluating the log2 ratio between the two conditions associated to a standard t-test [89], adjusted for multiple testing by the False Discovery Rate (FDR) approach [90]. All probe sets that differed more than to two-fold with a t-test P-value ≤ 0.05 were considered to be differentially expressed. The Significance Analysis of Microarrays (SAM) two-class unpaired analysis [91] was also performed in order to identify a more extensive list of differentially expressed genes, with the measure significant fold change set at 2.0 and a false discovery rate <8.4%. The expected proportion of significantly different features (p0) was set to 0.95.

### Data analysis

Functional categories significantly associated (P-value ≤ 0.05, adjusted using the FDR correction) with the up- and down-regulated sequences were identified using GeneBins, a database that provides a hierarchical functional classification modelled on the KEGG ontology [92] of probe set sequences represented on Affymetrix arrays [40]. We used PathExpress [93], a web-based tool based on the KEGG Ligand database [94], to detect whether probe sets associated with a metabolic pathway or sub-pathway were statistically over-represented in the differentially expressed sets of sequences (P-value ≤ 0.05). In addition, probe sets of the Affymetrix Medicago Genome Array were assigned to gene families described in the TAIR database [95] and to transcription factor families provided by the Database of Arabidopsis Transcription Factors [43] based on their sequence similarity with *Arabidopsis thaliana *proteins. Blastx [96] was used to find the best match (E-value ≤ 10^-8^) for the sequences representing each probe set (i.e. sequences derived from the most 5' to the most 3' probe in the public UniGene cluster). The differentially expressed sets of sequences were compared to the composition of each gene family to identify if a certain category was statistically over-represented. For each test, a P-value, representing the probability that the intersection of the list of up- or down-regulated probe sets with the list of probe sets belonging to the given gene family occurs by chance, was calculated using the hypergeometric distribution [97].

### Sequence analysis

Sequences of interest were analysed using BLAST and multiple sequence alignments to identify genes and proteins with sequence similarity from Arabidopsis. To identify orthologs in Arabidopsis, AffyTrees was used . AffyTrees automatically detects sequence orthologs based on phylogenetic trees.

### Comparing to Medicago Expression Atlas

To identify common genes expressed between embryogenic cultures and developing seeds, we have compared our data to that of the Medicago Expression Atlas [44]. We have chosen seed10d (Developing seeds at early embryogenesis – 10 days after pollination) since it is the earliest time point for seed development available in the Atlas and contrasted this to leaf (4-week old trifolia that were harvested without their petioles but with their petiolule) and have computed the average between all replicates, ratios (seed/leaf), log2 (ratio), t test adjusted with FDR method). Then we compared these lists with our data to see any overlap.

### Real-time RT-PCR

Total RNAs were isolated from the proliferating leaf explant cultures of *M. truncatula *line 2HA and Jemalong using the Qiagen RNeasy plant mini kit (Qiagen) and the total RNA was treated in 1× buffer with 2 U of DNAse I (Ambion, Austin, TX, USA) added to the reaction and incubated for 30 min at 37°C. The reaction was stopped by adding DNase Removal Reagent (Ambion). cDNA synthesis was done using 2 μg total RNA. One microliter of 5 μM oligo dT18 primer (5'-TTT/TTT/TTT/TTT/TTT/TTT-3') was added to the reaction, and incubated for 10 min at 70°C, then chilled on ice. First strand mix containing 1× buffer, 10 mM DTT, 1.25 mM of each dATP, dCTP, dTTP, dGTP, was added to a total volume of 20 μL and incubated for 5 min at 42°C. Then 200 U SuperScript™ III reverse transcriptase (Invitrogen, Carlsbad, California, USA). For the no reverse transcriptase control, water was added instead of SuperScript III reverse transcriptase. The reaction was stopped by incubating at 70°C for 15 min and the final reaction either stored at -20°C or used for PCR immediately. For the real-time reverse transcription polymerase chain reaction (RT-PCR), gene specific primers (Table 3) were designed using Primer Express software (Applied Biosystems) and ordered from Sigma Genosys (Castle Hill, NSW, Australia). The PCR was carried out in a total volume of 10 μL containing 0.3 μM of each primer, 1× SYBR green PCR master mix (PE Applied Biosystems). Reactions were amplified as follows: 95°C for 10 min, then 40 cycles of 95°C for 15 sec, 60°C for 1.5 min. Amplifications were performed in 384-well clear optical reaction plates (Applied Biosystems) with an ABI PRISM 7900 Sequence Detection System (at the Biomolecular Resource Facility, JCSMR, ANU) using version SDS 2.2.2 software (Applied Biosystems) to analyse raw data. The absence of genomic DNA and non-specific by-products of the PCR amplification was confirmed by analysis of dissociation curves and agarose gel electrophoresis of the PCR products (data not shown). The gels were stained with 0.5 μg mL^-1 ^ethidium bromide, visualised using an UV transilluminator and then photographed. Normalisation was done as described [46] using MtUBQ10 (Ubiqutin10, TC100142) as a control gene. Three biological repeats (independent tissue culture experiments performed in parallel under same growth condition) were done for each treatment.

**Table 3:** Primers used in real-time RT-PCR assay

Probe ID	Accession number	Description	Forward primers	Reverse primers
Mtr.47631.1.S1_s_at	1645.m00036	Transposase	5'-CGTTACCCTGTTTTGGCAACA-3'	5'-GCTCTCCGAAGCAACTGATGA-3'
Mtr.15107.1.S1_at	775.m00015	RNA-directed DNA polymerase	5'-CCAATTGATAAAAGTGGTGCAAAT-3'	5'-TGACTCCCTTTGATCCTGTAGCT-3'
Mtr.45925.1.S1_s_at	740.m00009	GH	5'-TGACTGAAACGTTAACTGGAGTAAGG-3'	5'-TTTCAAAGGTAATCCTCCTAGCAAA-3'
Mtr.43735.1.S1_at	TC95950	MtRR1	5'-TGAAACGGAGCTGGTGATGA-3'	5'-CAATCTCACAGGTTTCAGCAGAA-3'
Mtr.47174.1.S1_at	1693.m00050	AGL20	5'-AGAACAGCAGTTGGAAAAGAGTGTT-3'	5'-TTTTAGTTGGTCAATTTGATGCTTGT-3'
Mtr.41073.1.S1_at	TC108662	FPF1	5'-TTGGTCGAGAATCCTCAAGCA-3'	5'-TGTGGGCAAGTGAACCAACA-3'
Mtr.8427.1.S1_at	TC100141	LipOx	5'-AGCCTTGGTGGCCTGAGAT-3'	5'-CGGATGCAAGCCATATGATTATT-3'
Mtr.10439.1.S1_at	TC106784	EIN3	5'-CGATTAAAGGAGCAAGTCAAACC-3'	5'-TTGCCTGTTCCTGGGATTG-3'
Mtr.8585.1.S1_at	TC100726	MtN3	5'-TGATGTTGTGAAGATTGGAACAGA-3'	5'-TGGATCCCATGTTAAAATCAGACTT-3'
Mtr.18380.1.S1_at	949.m00022	Fasciclin	5'-CCTAGTGATTCCACCCCTGACA-3'	5'-GCCTTCGCCTTTCTCAGGAT-3'
Not on the array	TC100142	Ubiqutin10	5'-GAACTTGTTGCATGGGTCTTGA-3'	5'-CATTAAGTTTGACAAAGAGAAAGAGACAGA-3'

Accession numbers starting with TC are from TIGR gene Index (MtGI) otherwise from the annotation by the International Medicago Genome Annotation Group (IMGAG). GH, *Glycoside hydrolase*; *RR*, response regulator; *AGL20*, agamous-like 20; *FPF*, flower promoting factor; *LipOx*, lipoxygenase; *EIN*, ethylene-insensitive; *MtN3*, *Medicago truncatula *nodulin3.

**Table 4:** Transcription factor families that are different between the embryogenic and the non-embryogenic cultures.

**Family**	**Number of probe sets on array**	**≥ 2 fold up in embryogenic culture**	**≥ 2 fold up in non-embryogenic culture**
AP2/EREBP	140		1 (p = 0.126)
AUX/IAA	25		1 (p = 0.024)
bHLH	277	5 (p = 0.005)	
C2C2-co-like	39	1 (p = 0.140)	
C2C2-DOF	274	1 (p = 0.654)	
C2H2	199	2 (p = 0.179)	2 (p = 0.016)
C3H	169	1 (p = 0.480)	
EIL	6		1 (p = 0.006)
GARP-ARR-B	19	1 (p = 0.071)	
GRAS	75	1 (p = 0.251)	
MADS	54	1 (p = 0.188)	
MYB	209	2 (p = 0.193)	
WRKY	837	6 (p = 0.106)	1 (p = 0.556)

Transcription factors were predicted by homology relationship based on the Database of Arabidopsis Transcription Factors and grouped by families.

## Authors' contributions

NI and MN conducted all experiments and drafted the manuscript. NG performed statistical and bioinformatics analysis. NI and BGR participated in the design of the study. NI wrote the manuscript.

## Supplementary Material

Additional file 1 **The list of differentially expressed genes identified by the Significance Analysis of Microarrays (SAM) two-class unpaired analysis**[43]. Fold change threshold was set at 2.0 and a false discovery rate was <8.4%. The expected proportion of significantly different features (p0) was set to 0.95. Click here for file

Additional file 2 **Venn diagram showing overlap between the two-fold cut-off method and SAM two-class unpaired analysis.** Click here for file

Additional file 3 **Microarray normalised data and expression ratios for the *M. truncatula *super-embryogenic culture (2HA) and the non-embryogenic culture Jemalong (Jem).** All quantitative data is expressed as log2 (embryogenic: non-embryogenic) expression ratios. Click here for file

Additional file 4 **Gene family classification for transcripts ≥ 2.0 fold differentially expressed.** Click here for file

Additional file 5 **Transcription factors ≥ 2.0 fold differentially expressed, as predicted by homology relationship based on members of Database of Arabidopsis Transcription Factors.** Click here for file

Additional file 6 Comparison of gene expression between the embryogenic cultures and developing seeds. Click here for file

Additional file 7 Venn diagram showing overlap between up- or down-regulated genes between the embryogenic cultures and developing seeds. Click here for file
