# Supplementary material for: Genome-wide transcriptional analysis of super-embryogenic Medicago truncatula explant cultures
Source: BMC Plant Biol. 2008 Oct 27;8:110. doi: 10.1186/1471-2229-8-110 (PMC2605756; doi:10.1186/1471-2229-8-110)
Supplement: Additional file 2 — Venn diagram showing overlap between the two-fold cut-off method and SAM two-class unpaired analysis. [file 1471-2229-8-110-S2.doc]

195

Fold-change/*t* test

SAM

Up-regulated in embryogenic cultures

38

Down-regulated in embryogenic cultures
