# Supplementary material for: Genome-wide transcriptional analysis of super-embryogenic Medicago truncatula explant cultures
Source: BMC Plant Biol. 2008 Oct 27;8:110. doi: 10.1186/1471-2229-8-110 (PMC2605756; doi:10.1186/1471-2229-8-110)
Supplement: Additional file 7 — Venn diagram showing overlap between up- or down-regulated genes between the embryogenic cultures and developing seeds. [file 1471-2229-8-110-S7.doc]

**2HA/Jemalong**

**Seed/Leaf**

**96**

Differentially expressed

**14**

Up-regulated/Up-regulated

**6**

Down-regulated/Down-regulated

**7**

Down-regulated/Up-regulated

Up-regulated/Down-regulated

**69**
